# ZHP-3 Acts at Crossovers to Couple Meiotic Recombination with Synaptonemal Complex Disassembly and Bivalent Formation in *C. elegans*

**DOI:** 10.1371/journal.pgen.1000235

**Published:** 2008-10-24

**Authors:** Needhi Bhalla, David J. Wynne, Verena Jantsch, Abby F. Dernburg

**Affiliations:** 1 Department of Molecular and Cell Biology, University of California Berkeley, Berkeley, California, United States of America; 2 Life Sciences Division, E. O. Lawrence Berkeley National Lab, Berkeley, California, United States of America; 3 Department of Chromosome Biology, Max F. Perutz Laboratories, University of Vienna, Vienna, Austria; Stowers Institute for Medical Research, United States of America

## Abstract

Crossover recombination and the formation of chiasmata normally ensure the proper segregation of homologous chromosomes during the first meiotic division. *zhp-3*, the *Caenorhabditis elegans* ortholog of the budding yeast *ZIP3* gene, is required for crossover recombination. We show that ZHP-3 protein localization is highly dynamic. At a key transition point in meiotic prophase, the protein shifts from along the length of the synaptonemal complex (SC) to an asymmetric localization on the SC and eventually becomes restricted to foci that mark crossover recombination events. A *zhp-3::gfp* transgene partially complements a null mutation and reveals a separation of function; although the fusion protein can promote nearly wild-type levels of recombination, aneuploidy among the progeny is high, indicating defects in meiotic chromosome segregation. The structure of bivalents is perturbed in this mutant, suggesting that the chromosome segregation defect results from an inability to properly remodel chromosomes in response to crossovers. *smo-1* mutants exhibit phenotypes similar to *zhp-3::gfp* mutants at higher temperatures, and *smo-1*; *zhp-3::gfp* double mutants exhibit more severe meiotic defects than either single mutant, consistent with a role for SUMO in the process of SC disassembly and bivalent differentiation. We propose that coordination of crossover recombination with SC disassembly and bivalent formation reflects a conserved role of Zip3/ZHP-3 in coupling recombination with SC morphogenesis.

## Author Summary

Sexual reproduction relies on meiosis. This specialized cell division generates gametes, such as sperm and eggs, with a single copy of the genome, so that fertilization restores diploidy. In order for chromosomes to segregate correctly during meiosis, homologs usually must undergo crossing over (genetic exchange) during meiotic prophase. How crossovers are coupled to large-scale changes in chromosome structure is not well understood. Our work shows that the protein ZHP-3 localizes to crossovers late in prophase, coincident with a transition in which chromosomes initiate progressive restructuring around the crossover. We have found that a ZHP-3-GFP fusion protein is competent to promote genetic exchange but not proper segregation. Chromosomes from these mutant animals exhibit defects in this late-prophase restructuring, suggesting that alterations in chromosome architecture that typically accompany crossovers have not occurred. We propose that ZHP-3 acts at crossovers to coordinate genetic exchange with higher order changes in chromosome structure that promote proper chromosome segregation.

## Introduction

Meiosis generates haploid gametes from diploid cells by coupling a single round of replication with two successive chromosomes segregation events: meiosis I, in which homologous chromosomes segregate away from each other and meiosis II, in which sister chromatids are partitioned. To ensure proper homolog disjunction, physical linkages must be introduced between homologs by processes that occur during meiotic prophase: homolog pairing, the assembly of the synaptonemal complex (SC), and crossover recombination. Together these mechanisms introduce the chiasmata that link homologs together until anaphase I. Defects in any of these processes produce meiotic chromosome segregation defects that lead to inviability among the resulting zygotes, and can also result in developmental defects and cancer predisposition.

In budding yeast, the Zip3 protein appears to couple crossover recombination and synapsis [1]. It is a member of both the ZMM class of proteins, required for the commitment to crossover formation [2], and the synapsis initiation complex (SIC), a group of proteins that localize to sites of stabilized homolog pairing to initiate SC assembly [3]. Zip3 and its orthologs in other species contain a RING domain, suggesting that the protein may have ubiquitin or SUMO (small ubiquitin-related modifier) ligase activity [4]. RING fingers are usually found in E3 ligases, which act in concert with E1 and E2 enzymes to covalently attach these small polypeptides to target proteins to modify their function, localization, and/or stability [5]. Zip3 promotes the formation of SUMO polymeric chains *in vitro* [6], and SUMO conjugation has been implicated in synapsis regulation [7]. Zip1, a structural element of the SC, has affinity for SUMOylated proteins [6]. One hypothesis is that SUMOylation of targets on paired but unsynapsed chromosomes may regulate SC assembly so that it only occurs between properly paired homologs [8]. A non-null mutation in the sole SUMO E2 ligase gene in budding yeast, *UBC9*, affects recombination less severely than mutation of *ZIP3*, suggesting that the putative SUMO ligase activity of Zip3 might be required for synapsis but not for crossover formation [7]. Mutations that abrogate Zip3's SUMO ligase activity exhibit defects in SC assembly and spore viability, but crossover formation has not been directly assessed in these mutants [6].

In the budding yeast *S. cerevisiae*, recombination and synapsis are obligately linked, but the nematode worm *C. elegans* assembles SC between homologous chromosomes efficiently in the absence of recombination [9]. Worms lacking *zhp-3*, the *C. elegans* homolog of *ZIP3*, accomplish homologous synapsis and initiate recombination, but fail to form crossovers. The persistence of early DSB break intermediates in this mutant supports a role for the *C. elegans* gene early in the recombination process [10]. A ZHP-3-GFP fusion protein expressed from a high-copy-number transgene array localizes to the SC independently of SPO-11, the conserved endonuclease that introduces programmed double strand breaks (DSBs) necessary for the initiation of recombination. Localization of this fusion protein depends on SC assembly [10], consistent with the finding that synapsis is a requirement for crossover formation in this organism [11]–[13].

During the diplotene stage, shortly before metaphase I, the synaptonemal complex begins to disassemble, and homologs undergo transient decondensation. In *C. elegans*, SC disassembly occurs asymmetrically, with loss of central element proteins along one “arm” of each chromosome pair. This asymmetry is thought to be directed by sites of crossover recombination, and to guide the subchromosomal localization of components, including the aurora kinase AIR-2, which regulate the orderly release of cohesion and proper chromosome segregation [14]. Concomitant with or soon after desynapsis, chromosome arms condense and re-orient around the site of a crossover to form a compact, cruciform bivalent. The condensin complex, a regulator of mitotic and meiotic chromosome condensation, promotes aspects of this restructuring [15]. However, many details of this dynamic restructuring remain poorly understood.

The work presented here implicates ZHP-3 as an important player in these events in *C. elegans*. We find that the protein exhibits dynamic localization during meiotic prophase, localizing along the SC in early pachytene, asymmetrically on the SC in late pachytene, and at foci in late pachytene/diplotene. These foci number six per nucleus in wild-type hermaphrodites and mark the boundary of asymmetric SC disassembly during diplotene/diakinesis. Analysis of mutants that exhibit perturbed number and placement of crossovers further supports the idea that ZHP-3 foci correspond to sites of reciprocal exchange.

We have found that a ZHP-3-GFP fusion protein expressed from a low-copy-number transgene that has been stably integrated into the genome (*zhp-3::gfp*) largely recapitulates the localization of endogenous ZHP-3 and partially complements a *zhp-3* null allele in a temperature-sensitive manner. Mutant animals expressing only ZHP-3-GFP produce elevated numbers of inviable embryos and male progeny, both indicative of meiotic chromosome missegregation. Cytological analysis indicates that oocytes expressing the fusion protein in the absence of wild-type ZHP-3 show defects in SC disassembly and bivalent structure, which likely account for their segregation defects. Nevertheless, this fusion protein promotes reciprocal exchange at nearly wild-type frequencies in the absence of endogenous ZHP-3. Together these observations suggest that crossovers are not sufficient to ensure proper homolog segregation, and that ZHP-3 performs an essential function after crossover formation which is compromised in the fusion protein. A mutation in the gene encoding the SUMO polypeptide, *smo-1*, results in defects resembling those seen in *zhp-3::gfp* mutants at higher temperatures, and exacerbates meiotic defects when combined with the *zhp-3::gfp* allele.

We propose that ZHP-3 has two separable roles during *C. elegans* meiosis, promoting crossover formation and mediating the appropriate restructuring of bivalents so that chiasmata ensure proper segregation. We suggest that this second role reflects a conserved function of the protein in coordinating recombination with synaptonemal complex morphogenesis; in contrast to its *S. cerevisiae* counterpart, Zip3, which appears to couple crossover recombination with synaptonemal complex assembly, *C. elegans* ZHP-3 coordinates recombination with SC disassembly and bivalent formation.

## Results

### ZHP-3 Localization Is Dynamic during Meiotic Prophase

**Figure 1:**
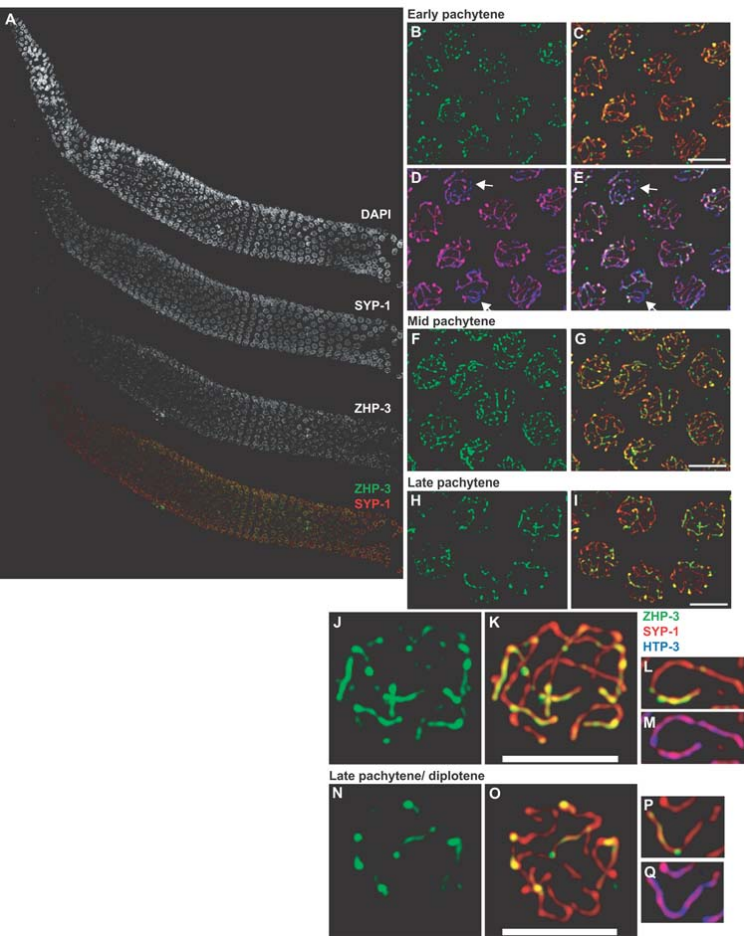
ZHP-3 shows dynamic localization during meiotic prophase. (A) Wild-type gonad stained with DAPI and antibodies against SYP-1 and ZHP-3. ZHP-3 largely colocalizes with SYP-1 throughout the gonad. High magnification views of meiotic nuclei in early pachytene (B, C, D and E), mid-pachytene (F and G) and late pachytene (H and I) stained with antibodies against ZHP-3, SYP-1 and HTP-3. The arrows in D and E indicate unsynapsed chromosomes, detected as segments of HTP-3 devoid of SYP-1 and ZHP-3. High magnification views of individual nuclei in late pachytene (J and K) and late pachytene/diplotene (N and O). A single pair of synapsed homologs from each of these nuclei is shown in L, M, P and Q. Note the asymmetric distribution of ZHP-3 in L and focus formation in P on synapsed chromosomes (M and Q). All images are maximum intensity projections of deconvolved 3D stacks. Scale bars represent 4 microns in all figures unless indicated otherwise.

The localization of ZHP-3 was previously analyzed using a GFP fusion protein expressed from a high-copy array integrated into the genome [10]. To understand its role in more detail, we generated polyclonal antibodies to localize the endogenous protein. The spatial and temporal organization of meiotic nuclei in the worm germline facilitates analysis of protein localization as a function of progression through meiotic prophase (see Figure 1A). During early prophase, we observed that ZHP-3 protein localized along the synaptonemal complex (SC), similar to previous observations. The protein was initially detected on chromosomes in early pachytene. Although its localization to the SC required the loading of central element components, as previously reported [10], it initially appeared as puncta along continuous stretches of SC (Figure 1B, C and E). This preceded complete synapsis, since ZHP-3 localized to synapsed chromosome regions in the presence of unsynapsed chromosomes, which did not stain with ZHP-3 (arrows in Figure 1D and E). ZHP-3 then accumulated along the SC so that by mid-pachytene, it had spread along the full length of the SC. However, by contrast to the axial and central element proteins (respectively) HTP-3 and SYP-1, its appearance was not uniform along the length of the SC (Figure 1F, G and data not shown). By late pachytene, localization along the length of the SC became more restricted, such that each synapsed homolog pair showed an extensive stretch of SYP-1 lacking ZHP-3 (Figure 1H–K). Three-dimensional reconstruction of pairs of homologs revealed that ZHP-3 was asymmetrically localized from an interstitial point to one end of the SC (Figure 1L). The presence of SYP-1 along the full length of these synapsed chromosomes (Figure 1L and M) indicates that this shift in ZHP-3 localization precedes the crossover-dependent asymmetric disassembly of the SC that accompanies the transition from late pachytene to diplotene [14]. Mutations that prevent complete synapsis and thereby extend the region of polarized nuclear morphology (*e.g.*, pairing center deficiencies or *him-8* mutants) also delayed the shift in ZHP-3 distribution, extending the region of nuclei that retain ZHP-3 localization along the length of the SC (data not shown).

**Figure 2:**
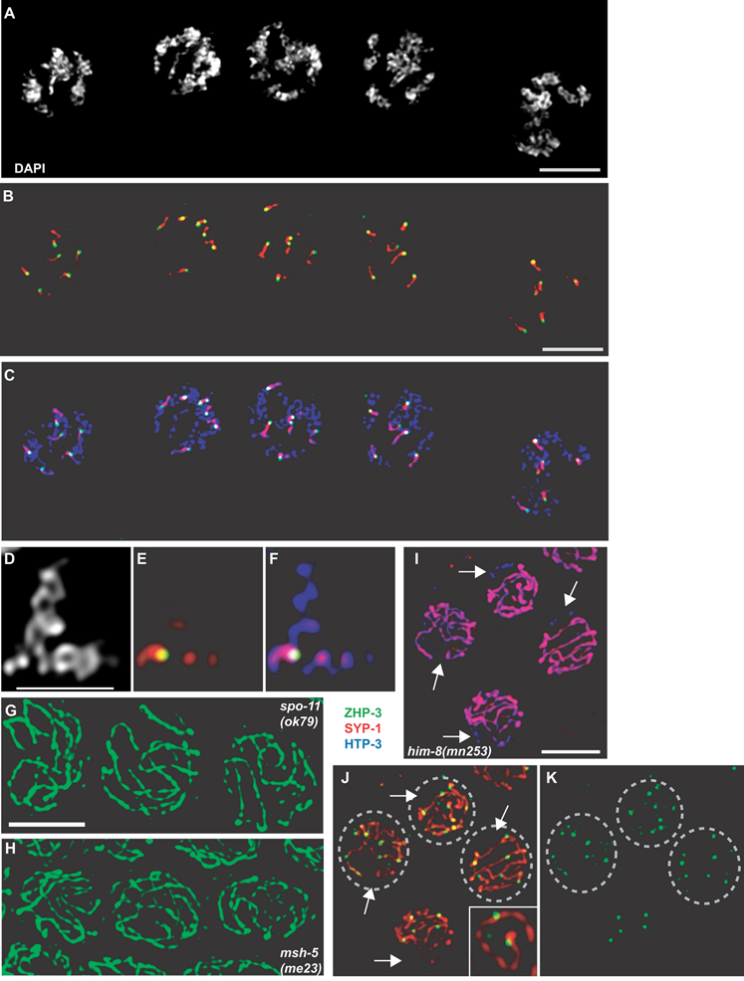
ZHP-3 localizes to sites of meiotic crossover recombination. (A–F) The site from which the asymmetric disassembly of the SC occurs is marked by a ZHP-3 focus. Wild-type nuclei in diplotene/diakinesis stained with DAPI and antibodies against HTP-3, ZHP-3 and SYP-1. An individual bivalent from one of these nuclei is shown in D–F. Scale bar in D represents 2 microns. (G and H) ZHP-3 foci formation depends on DSB formation and crossover recombination. Late pachytene nuclei from *spo-11(ok79)* (G) and *msh-5(me23)* (H) mutants were stained with antibodies against ZHP-3. (I, J, and K) More than one ZHP-3 focus per chromosome is frequently observed in mutant oocytes with perturbed crossover control. Late pachytene nuclei from *him-8(mn253)* mutants were stained with ZHP-3, SYP-1 and HTP-3 antibodies. Arrows indicate unsynapsed chromosomes. Circled nuclei have greater than five ZHP-3 foci. The inset in J is a single pair of synapsed homologs with two ZHP-3 foci.

In late pachytene and early diplotene nuclei, ZHP-3 became largely restricted to a single prominent focus on each pair of homologs (Figure 1N–Q). During late pachytene, faint ZHP-3 immunofluorescence could still be observed along the SC (Figure 1P), but this disappeared upon the initiation of SC disassembly at diplotene. These foci corresponded to the boundary between the “long arm” of the bivalent, from which the central element protein SYP-1 was removed, and the “short arm,” which retained SYP-1 until diakinesis (Figure 2B and C, magnified in 2E and F, [14]).

*C. elegans* chromosome pairs usually undergo only one crossover recombination event during meiosis [16]–[18], and the formation of these crossovers directs the asymmetric disassembly of the SC observed in diplotene/diakinesis [14]. It therefore seemed likely that ZHP-3 foci might correspond to meiotic crossovers. To test this possibility, we monitored ZHP-3 localization in meiotic mutants defective in crossover formation. SPO-11 is a conserved endonuclease required for programmed double strand breaks (DSBs), which are essential for meiotic recombination [19]. MSH-5 is a meiosis-specific homolog of the mismatch repair protein, MutS, which is required (along with its partner HIM-14/Msh4) downstream of DSBs to promote crossover formation [20],[21]. In *spo-11* and *msh-5* mutants, we observed persistence of ZHP-3 along the full length of the SC until diplotene (Figure 2G and H). In the absence of recombination, SC disassembly is dysregulated: some chromosomes lose SYP-1 staining altogether, some retain it along their length, and others are associated with brightly staining SYP-1 foci [14]. In such cases, we found that ZHP-3 remained associated with stretches of SYP-1 in diplotene, although some regions of SYP-1 without ZHP-3 could be observed (data not shown). However, ZHP-3 was no longer detected at diakinesis, even on univalents that retained SYP-1 (data not shown).

As further evidence that ZHP-3 foci mark crossovers, we detected perturbed patterns of protein localization in mutants for which such crossover defects have been demonstrated genetically. In *C. elegans*, crossover control is manifested in both the number and placement of crossovers [16]–[18],[22]. In *him-8* mutant hermaphrodites, asynapsis of the sex chromosomes results in a measurable increase in crossovers on autosomes [23],[24]. If crossover control were maintained in these animals, we would expect to see five ZHP-3 foci in each nucleus, corresponding to a single crossover event on each autosome. Consistent with genetic evidence, *him-8* oocytes (Figure 2I) at late pachytene often displayed more than five ZHP-3 foci (circled nuclei in Figure 2J and K), and we could detect individual pairs of synapsed chromosomes with more than one ZHP-3 focus (Figure 2J, inset). We did not observe ZHP-3 foci on unsynapsed chromosomes, consistent with evidence that synapsis is a prerequisite for ZHP-3 localization ([10] and Figure 1E). We also observed altered ZHP-3 localization in *rec-1* mutant hermaphrodites, which lack the normal crossover bias towards the ends of each autosome [25],[26]. In *rec-1* oocytes, we observed seven out of sixteen synapsed homolog pairs with a ZHP-3 focus near the center of the chromosome (Figure S1B). By contrast, four of five synapsed homolog pairs from wild-type animals had a ZHP-3 focus closer to one end (Figure S1A).

### A ZHP-3-GFP Fusion Protein Recapitulates Endogenous ZHP-3 Localization

**Figure 3:**
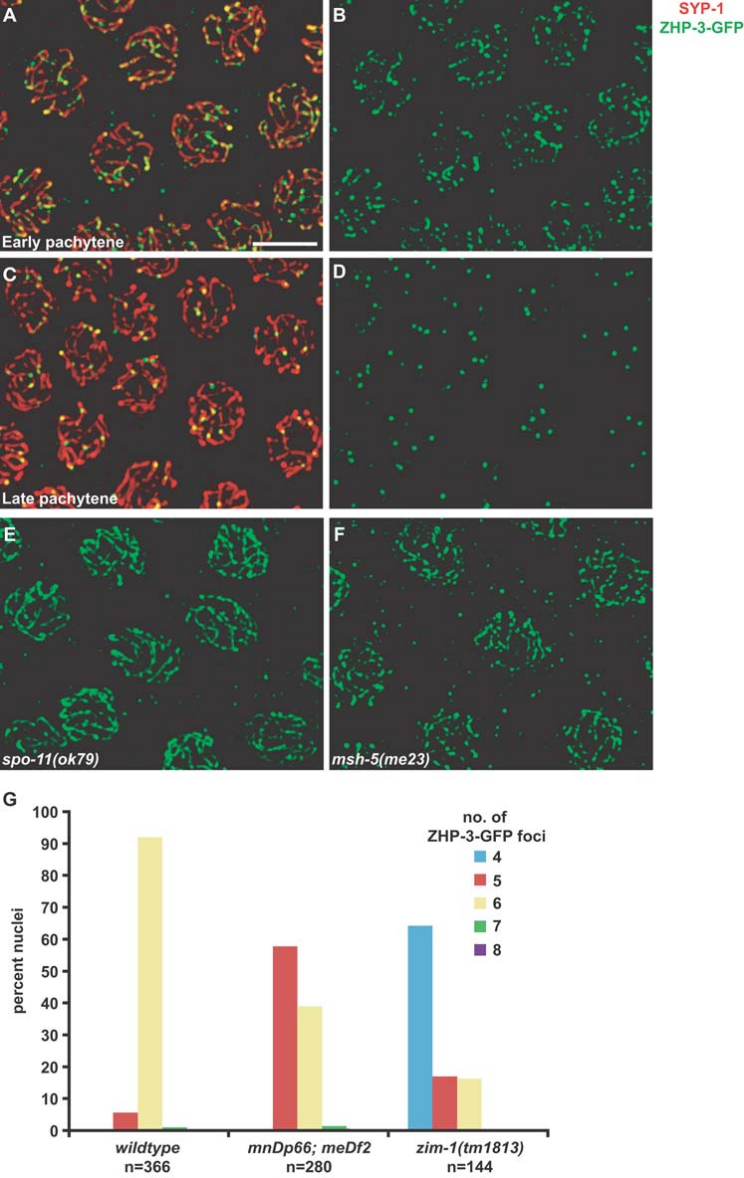
ZHP-3-GFP recapitulates the localization pattern of endogenous ZHP-3. Wild-type oocytes at early pachytene (A and B) and late pachytene (C and D) stained with antibodies against SYP-1 and GFP. (E and F) Formation of ZHP-3-GFP foci depends on double-strand breaks and crossover recombination. Late pachytene nuclei from *spo-11(ok79)* (E) and *msh-5(me23)* (F) mutants were stained with antibodies against GFP. G. Crossover distributions can be analyzed by direct visualization of ZHP-3-GFP. Numbers of ZHP-3-GFP foci were quantified in late pachytene nuclei of wild-type, *mnDp66*; *meDf2*, and *zim-1(tm1813)* animals.

A *zhp-3::gfp* fusion transgene driven by the germline *pie-1* promoter (*P_pie-1_::zhp-3::gfp*) was integrated into the worm genome by ballistic transformation. A previous ZHP-3-GFP transgene reported by Jantsch *et. al.* [10] had been introduced by integration of an extrachromosomal array, resulting in multiple copies of the transgene in the genome. By contrast, the ballistic transformation technique often results in transgenic strains with insertions at low-copy number, allowing the transgene to escape germline silencing and to more reliably recapitulate normal expression [27]. We observed that the encoded ZHP-3-GFP fusion protein localized to meiotic chromosomes in a pattern closely resembling that of the endogenous protein, as detected by immunofluorescence. It appeared on the SC in early pachytene (Figure 3A and B) and became more restricted later in prophase (Figure 3C and D). However, the foci at late pachytene appeared more prominent when the fusion protein was detected by anti-GFP antibodies (Figure 3D) than by immunofluorescence against the endogenous protein (Figure 1H). When we performed immunofluorescence in animals carrying the transgene with antibodies against both endogenous ZHP-3 and GFP, we observed colocalization (data not shown).

We observed six foci in 92.3% of meiotic nuclei in otherwise wild-type hermaphrodites carrying the *zhp-3::gfp* transgene, similar to the percentage observed in wild-type oocytes stained with anti-ZHP-3 antibody (95.5%). Furthermore, appearance of these foci was dependent on the functions of *spo-11* (Figure 3E) and *msh-5* (Figure 3F), and at diplotene they marked the boundaries between zones of SYP-1 retention and removal on all six chromosomes (data not shown).

The differences we observed in the localization patterns of the endogenous protein and the ZHP-3-GFP fusion protein raised the possibility that the fusion protein might not be fully functional, an issue we explored in greater detail (see below). We established that the trangene does not result in meiotic defects in animals carrying either one or two copies of the endogenous gene. Hermaphrodites expressing both the endogenous gene and the transgene produced broods indistinguishable from wild-type animals in number, embryonic viability and frequency of male self-progeny at 15°, 20° and 25°C (Table 1). This indicates that 1) the fusion protein does not result in a dominant negative effect at any temperature, and 2) expression of ZHP-3 from the *pie-1* promoter does not interfere with embryonic development. These properties, together with the prominence of ZHP-3-GFP foci at late pachytene, make the fusion protein a neutral and valuable cytological reporter for crossover formation when the endogenous gene product is also present (compare Figures 1G with 3D). This marker is particularly useful in the presence of mutations that alter meiotic progression or germline apoptosis, since these situations tend to have smaller populations of nuclei at the transient stage where foci can be measured reliably by immunofluorescence against the endogenous protein [23],[28],[29].

**Table 1:** *zhp-3::gfp* does not have a dominant effect.

	*wild-type*		*zhp-3(+)*; *P_pie-1_::zhp-3::gfp*	
	% viability (no. of progeny scored)	% male self progeny	% viability (no. of progeny scored)	% male self progeny
15°C	100% (3025)	0.1%	100% (3327)	0.2%
20°C	100% (3165)	0.2%	100% (3503)	0.1%
20°C	100% (2110)	0.1%	100% (1981)	.02%

We directly compared the ability of the endogenous ZHP-3 protein and the GFP fusion to report on crossover distribution in a mutant with defective *X* chromosome synapsis. *meDf2* is a deficiency of the *X* chromosome Pairing Center region that strongly reduces recombination on the *X* chromosome but elevates autosomal crossing-over [23],[30]. Apoptosis of *meDf2* oocytes is also elevated due to activation of the DNA damage checkpoint [28]. Over 40% of *meDf2* pachytene nuclei contained greater than five ZHP-3-GFP foci when animals were propagated at 20°(n = 280; Figure 3G). This value was very similar to that obtained by quantification of endogenous ZHP-3 immunofluorescence: 38.5% of germline nuclei in these animals exhibited greater than five ZHP-3 foci. In this experiment, care was taken to limit analysis to late pachytene nuclei with prominent foci, and this is reflected in the small number of nuclei counted (n = 52). Importantly, whether the endogenous or fusion protein was used as a marker, this cytological assay revealed a higher frequency of multiple crossover events on autosomes than the 5.6% of chromosomes previously detected by SNP-based analysis of progeny [23]. This may indicate that nuclei with multiple crossovers are enriched among the population of oocytes that are eventually culled by apoptosis, or that multiple crossover events may frequently escape detection by SNP analysis due to their chromosomal distribution.

We also quantified ZHP-3-GFP foci at 20° in *zim-1* mutants, in which two pairs of autosomes fail to synapse [31]. This experiment allowed us to address two questions: 1) Does autosomal asynapsis result in an increased frequency of multiple crossovers on other chromosomes? and 2) Does a greater number of unsynapsed chromosomes result in a more severe defect in crossover control? Most nuclei (65%) showed four ZHP-3-GFP foci, but 17% and 16% of nuclei contained five and six ZHP-3-GFP foci, respectively (Figure 3G). This revealed that the ability of an unsynapsed chromosome to increase crossing-over on other chromosomes is not limited to situations in which *X* chromosomes are asynapsed, and the severity of the defect is similar whether one pair or two pairs of chromosomes are prevented from synapsing. Similar to *meDf2* mutants, 35% of oocytes in *zim-1* mutants had more than one crossover per synapsed chromosome pair. Interestingly, we rarely saw more than six ZHP-3-GFP foci in meiotic nuclei of either mutant analyzed: 2.14% in *meDf2* (1.78% with seven foci per nucleus and 0.36% with eight) and 1.38% in *zim-1* mutants (0.69% with seven foci per nucleus and 0.69% with eight), compared to 1.37% in wild-type hermaphrodites (all with seven foci per nucleus). This suggests the existence of a mechanism to constrain the total number of crossovers. Similar observations were made in *him-8* and *meDf2* mutants in which immunofluorescence against endogenous ZHP-3 was performed (data not shown).

### ZHP-3-GFP Partially Complements *zhp-3* Null Mutants in a Temperature-Sensitive Manner

We crossed the *zhp-3::gfp* transgene into *zhp-3* loss-of-function mutants (*zhp-3(jf61)*; *P_pie-1_::zhp-3::gfp*) to determine whether the fusion protein can substitute for wild-type ZHP-3. Hereafter, animals carrying only the GFP fusion will be referred to as *zhp-3::gfp*. We observed a temperature-sensitive, partial rescue of the meiotic defects associated with this null allele. While homozygous *zhp-3(jf61)* mutants produced only ∼1% viable progeny at all temperatures assayed (15°, 20° and 25°C) (Table 2 and [10]), the progeny of hermaphrodites expressing only ZHP-3-GFP were 42% viable at 15°, 12.6% viable at 20° and 2.3% viable at 25° (Table 2). Because transgene expression can be elevated at higher temperatures, we wondered whether the effects of temperature might be due to overexpression of the transgene. To test this, we analyzed animals carrying only a single copy of the transgene (*zhp-3(jf61)*; *zhp-3::gfp/+*). Compared to animals with two copies, the progeny of these animals showed similar levels of embryonic viability and male self-progeny at 20° and 25° (Table 2), indicating that the meiotic defects are not likely to reflect protein overexpression.

**Table 2:** *zhp-3::gfp* partially rescues the meiotic defect of *zhp-3* null mutants in a temperature-sensitive manner.

	*zhp-3(jf61)*		*zhp-3(jf61)*; *P_pie-1_::zhp-3::gfp*		*zhp-3(jf61)*; *P_pie-1_::zhp-3::gfp/+*	
	% viability (no. of progeny scored)	% male self progeny	% viability (no. of progeny scored)	% male self progeny	% viability (no. of progeny scored)	% male self progeny
15°C	1.1% (1650)	22.2%	42.0% (1770)	19.1%	not determined	not determined
20°C	1.3% (1434)	27.8%	12.6% (1561)	21.3%	6.97% (1736)	24.0%
25°C	1.4% (502)	28.6%	2.3% (298)	42.8%	2.7% (321)	33.3%

**Figure 4:**
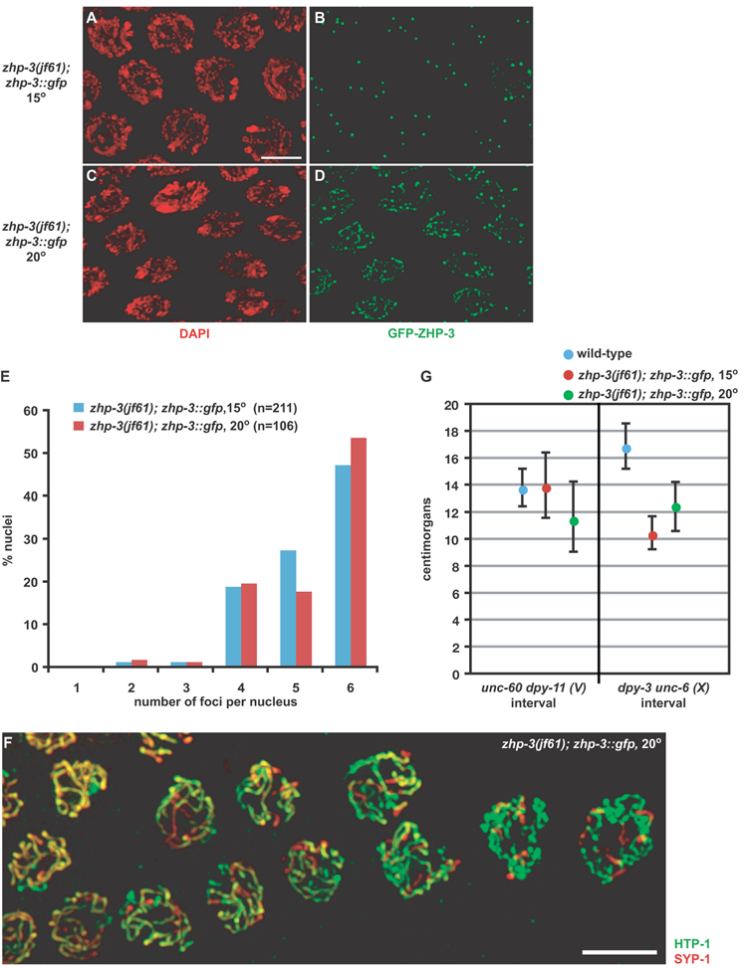
ZHP-3-GFP promotes crossover formation. (A–D) Localization of ZHP-3-GFP in *zhp-3::gfp* mutant animals at 15° and 20°. Late pachytene nuclei were stained with DAPI (A and C) and antibodies against GFP (B and D). Foci are visible at both temperatures. However, at the higher temperature, ZHP-3-GFP is also seen along the SC. (E) Quantification of ZHP-3-GFP foci in animals grown at 15° and 20°. The majority of nuclei contain five or six ZHP-3-GFP foci. Number of nuclei quantified for each genotype is indicated. (F) Asymmetric disassembly of the SC occurs normally in *zhp-3::gfp* mutants grown at 20°. Oocytes exiting pachytene are visualized with antibodies against HTP-1 and SYP-1. (G) Crossover frequencies in wild-type and *zhp-3::gfp* mutant hermaphrodites. Recombination in two different genetic intervals, one on Chromosome *V* and one on *X* were measured in wild-type hermaphrodites grown at 20° and mutant hermaphrodites grown at 15° and 20°. Error bars indicate 95% confidence intervals.

The increased nondisjunction seen in *zhp-3::gfp* hermaphrodites at elevated temperature could indicate that the fusion protein is unstable at high temperature. We tested this by localizing the ZHP-3-GFP fusion protein in the germlines of worms propagated at the different temperatures. To our surprise, not only did we detect similar levels of protein at all temperatures, but we also observed similar numbers of foci at late pachytene (Figure 4B, D and data not shown). At 15°, foci were visible in late pachytene meiotic nuclei, much as when the fusion protein is present along with the endogenous protein (Figure 4B). At 20° and 25°, ZHP-3-GFP foci were detectable but co-existed with stretches of ZHP-3-GFP along the length of the SC (Figure 4D and data not shown). Gonads from animals grown at higher temperatures also showed more extensive regions of nuclei with polarized morphology (Figure 4C), suggesting that meiotic progression may be perturbed in these situations despite complete synapsis (data not shown). We quantified foci in animals grown at 15° and 20°; this was not done at 25° because of the abundance of ZHP-3-GFP along the SC at this temperature. Most oocytes displayed five or six foci, with a mean number of 5.15 at both temperatures, suggesting that similar numbers of crossovers are completed at 15° and 20° (Figure 4E). Consistent with the interpretation that these foci represent functional crossovers, diplotene nuclei underwent asymmetric synaptonemal complex disassembly at all temperatures (Figure 4F and data not shown). Upon pachytene exit, SYP-1 and the axial element protein HTP-1 acquired reciprocal distributions, as in wild-type hermaphrodites [32]. This suggests that crossovers occur on most chromosomes in *zhp-3::gfp* mutants and that they are able to direct asymmetric disassembly of SC components.

To directly monitor whether ZHP-3-GFP foci reflect crossover formation in *zhp-3::gfp* mutants, we assayed recombination. Crossing-over was measured genetically for the *dpy-6 unc-3* interval, which spans most of the right half of the *X* chromosome, and the *unc-60 dpy-11* interval, spanning the left portion of Chromosome *V* (Figure 4G and Table 3). Consistent with our cytological observations, we detected wild-type levels of autosomal recombination in *zhp-3::gfp* mutant hermaphrodites grown at 15° (Table 3 and Figure 4G). At 20°, recombination on the autosome was slightly reduced (Table 3). However, this value was not significantly different than wild-type (Figure 4G) and is unlikely to explain the sharply reduced viability of progeny from animals maintained at this temperature. At 15° and 20°, we observed a statistically significant decrease in crossovers on the *X* chromosome (63% and 75% of wild-type levels, respectively) (Table 3 and Figure 4G). Therefore, the levels of embryonic inviability and incidence of males we observe in the progeny of *zhp-3::gfp* mutants do not result from an inability to introduce crossovers. Taken together with the striking localization of ZHP-3 on chromosomes in late prophase (Figures 1H–Q, 2A–F), this suggests that the protein likely plays an essential role in segregation after the completion of crossovers.

**Table 3:** The frequency of recombination in *zhp-3::gfp* mutants is similar to wild-type.

Genotype	Map Distance	Map distance
	*unc-3 dpy-6 (X)* interval	*unc-60 dpy-11 (V)* interval
	(no. of progeny scored) *(percent wild-type recombination)*	(no. of progeny scored) *(percent wild-type recombination)*
wild-type	16.7 (3211)	13.6 (2351)
	*(100%)*	*(100%)*
*zhp-3(jf61)*; *P_pie-1_::zhp-3::gfp*, 15°C	10.6 (1708)	13.9 (1078)
	*(63.3%)*	*(102%)*
*zhp-3(jf61)*; *P_pie-1_::zhp-3::gfp*, 20°C	12.5 (940)	11.4 (741)
	*(74.6%)*	*(83.9%)*

### Mutation of *smo-1* Results in Mislocalization of ZHP-3 in Late Pachytene and Genetically Interacts with the *zhp-3::gfp* Mutation

Bioinformatic analysis and in vitro biochemical data have indicated that Zip3, the yeast ortholog of ZHP-3, may be a SUMO ligase [4],[6]. We therefore explored the potential role of SUMOylation in ZHP-3 function. In *C. elegans*, as in *S. cerevisiae* and *Drosophila*, a single gene encodes the small ubiquitin-like modifier SUMO: *smo-1*. Inactivation of this gene by RNAi results in embryonic lethality [33],[34], indicating that the SUMO conjugation system is essential. The *C. elegans* gene knockout consortium has generated a deletion of the *smo-1* gene (*ok359*). Homozygous *smo-1* mutants from heterozygous mothers are viable, probably due to maternally supplied SMO-1 protein and/or mRNA, but the animals show defects in cell fate specification [35],[36]. Although they survive to adulthood, these animals are also sterile, in that they fail to produce mature eggs and sperm [35]; however, their gonads proliferate and their oocytes progress through meiotic prophase to the diakinesis stage.

**Figure 5:**
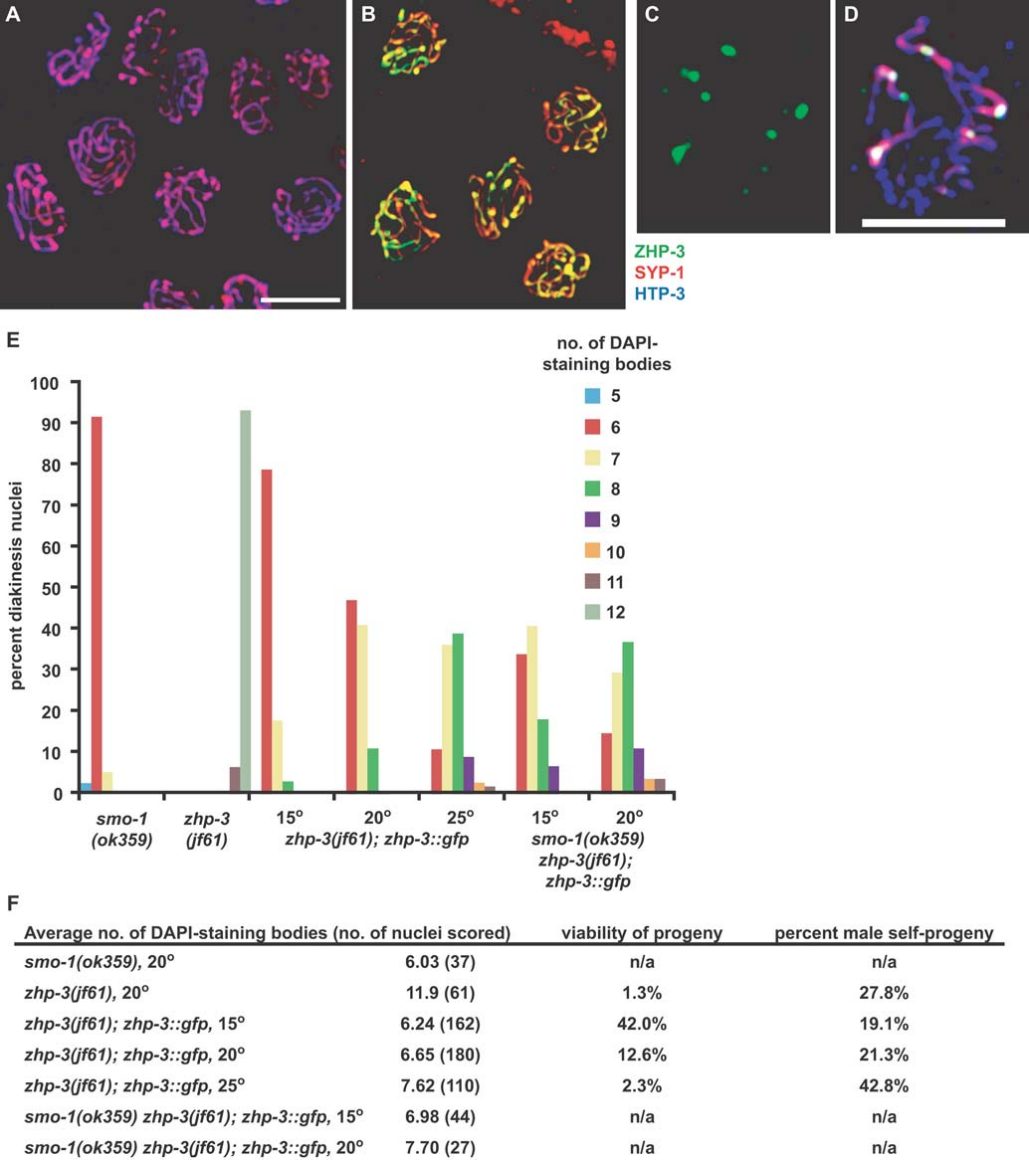
*smo-1(ok359)* genetically interacts with the *zhp-3::gfp* allele. (A) Synapsis occurs normally in *smo-1(ok359)* mutants. Late pachytene nuclei stained with antibodies against HTP-3 and SYP-1 are shown. (B) ZHP-3 fails to redistribute in late pachytene in *smo-1(ok359)* mutants. Nuclei were stained with antibodies against ZHP-3 and SYP-1. (C and D) ZHP-3 eventually forms six foci per nucleus during SC disassembly. Diplotene/diakinesis nuclei were stained with antibodies against ZHP-3, SYP-1 and HTP-3. (E) The number of univalents increases with temperature in *zhp-3::gfp* mutants. This defect is exacerbated by introduction of the *smo-1(ok359)* deletion into mutant animals. DAPI-staining bodies were scored in the indicated genotypes at 15°, 20° and 25°. (F) Average number of DAPI-staining bodies. To facilitate comparison, the percentage of viable progeny and male self-progeny generated by each genotype is indicated.

Synapsis did not appear to be perturbed in *smo-1* mutant animals (Figure 5A). However, ZHP-3 showed aberrant localization. In lieu of the normal asymmetric distribution along the SC (Figure 1H and I), ZHP-3 remained associated along the length of the SC in late pachytene (Figure 5B). This is similar to what we observed in *zhp-3::gfp* mutants at higher temperatures. In diplotene nuclei undergoing SC disassembly (Figure 5C and D), six ZHP-3 foci were observed, and most oocytes at diakinesis contained six DAPI-staining bodies (Figure 5E), indicating that *smo-1* mutants are competent to form crossovers. Virtually identical effects were observed when *ubc-9*, which encodes the E2 ligase required for the SUMO conjugation pathway [5], was disrupted by mutation (data not shown).

We observed a genetic interaction between the *smo-1* deletion and *zhp-3::gfp* allele. Because deletion of *smo-1* results in sterility, we could not assay embryonic viability or recombination. However, we observed that the number of DAPI-staining bodies at diakinesis was greater in *zhp-3::gfp*; *smo-1* double mutants than in *zhp-3::gfp* animals. Synaptonemal complex disassembly and bivalent differentiation are complete at this stage in meiotic prophase and chiasmata normally maintain a link between homologs. In wild-type hermaphrodites, six bivalents, corresponding to the six chromosome pairs, can normally be detected [9]. *zhp-3* loss-of-function mutants lack crossovers, and 12 univalent chromosomes were consequently detected in diakinesis nuclei ([10] and Figure 5E). Most nuclei in *smo-1* mutants displayed six DAPI-staining bodies at diakinesis (Figure 5E). At 15°, the *zhp-3::gfp* mutant exhibited six DAPI-staining bodies in most diakinesis nuclei (79%), while a fraction of nuclei contained seven (18%) or eight (3%) DAPI-staining bodies (Figure 5E). The mean number of DAPI-staining bodies at 15° was 6.24 (Figure 5F). At 20°C the mean number of DAPI-staining bodies increased to 6.65, and at 25°C it was 7.62 (Figure 5E and F). These differences are significant (p<0.0001 for each pairwise comparison). Deletion of *smo-1* in *zhp-3::gfp* animals resulted in exacerbation of this defect at 15° and 20° (Figure 5E and F) (p value<0.0001 for each pairwise comparison). *smo-1* animals fail to develop at 25° [35], precluding analysis at this temperature.

### Defective Bivalent Differentiation in *zhp-3::gfp* and *smo-1* Mutants

**Figure 6:**
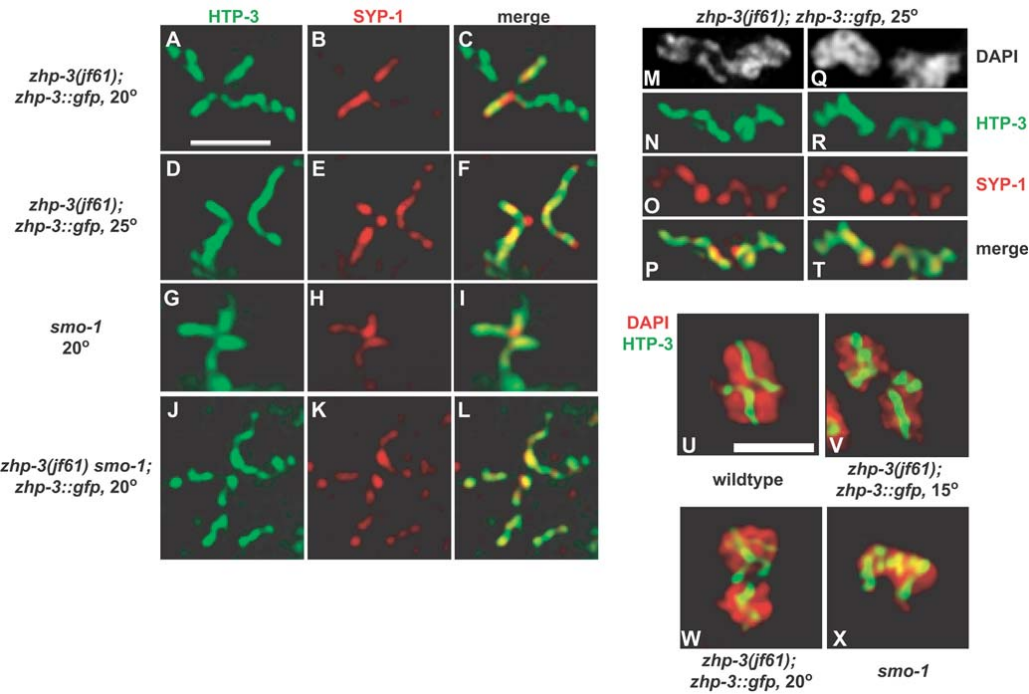
Defects in SC disassembly and bivalent structure in *zhp-3::gfp* mutants. (A–L) Synaptonemal complex disassembly is disrupted in *zhp-3::gfp* mutants propagated at 25° and in *smo-1* mutants. Bivalents in diakinesis nuclei were stained with antibodies against HTP-3 and SYP-1. At 20°, *zhp-3::gfp* mutants resemble wild-type animals in that SYP-1 is restricted to the short arm of the diakinesis bivalent. In *zhp-3::gfp* mutants at 25°, *smo-1* single mutants and *smo-1; zhp-3::gfp* double mutants at 20°, SYP-1 is seen all along the bivalent, similar to the axial element HTP-3. Scale bar indicates 2 microns. (M–P) As homologs desynapse in *zhp-3::gfp* mutants at 25°, SYP-1 is enriched along what will become the short arm of the bivalent, but is still visible on the long arm. (Q–T) Homologs sometimes appear to separate precociously at diplotene in *zhp-3::gfp* animals. (U–X) Bivalent structure is disrupted in *zhp-3::gfp* and *smo-1* mutants. Bivalents are stained with DAPI and antibodies against HTP-3. Scale bar indicates 2 microns.

Based on our findings that animals expressing *zhp-3::gfp* in the absence of endogenous ZHP-3 have mostly recombinant chromosomes but very high rates of meiotic nondisjunction, we looked for chromosome structural defects at diakinesis that might explain their segregation problems. We examined whether the distribution of SC components was altered at diakinesis in *zhp-3::gfp* mutants. Diakinesis bivalents in *zhp-3::gfp* mutants grown at 15° and 20° exhibited normal patterns of SYP-1 and HTP-3 staining: SYP-1 was limited to the short arm of the bivalent while HTP-3 stained both axes (Figure 6A–C and data not shown). However, in *zhp-3::gfp* mutants propagated at 25°, SYP-1 remained along both axes (Figure 6D–F), indicating abnormal SC disassembly. This does not occur in wild-type animals at 25° (data not shown). This defect is subtle in diplotene; SYP-1 was enriched on the short arms of desynapsing homolog pairs but still visible on the long arms (Figure 6M–P), and likely becomes more apparent along the long arm as the bivalent condenses. The retention of SYP-1 along the long arm at 25° may be a more severe manifestation of defects that also occur at lower temperatures but are not detectable by our assays (see below).

Similar perturbation of SC disassembly was observed in *smo-1* mutants maintained at either 15°and 20° (Figure 6G–I and data not shown), consistent with a role for SUMO in the disassembly process. In *smo-1*; *zhp-3::gfp* mutants propagated at 15° and 20°, the *smo-1* defect in SC disassembly was epistatic to the *zhp-3::gfp* phenotype at these temperatures (Figure 6J–L and data not shown).

The appearance of chromosomes undergoing SC disassembly and bivalent differentiation in *smo-1* and *zhp-3::gfp* mutants did not resemble the typical X or Y shape observed in wild-type animals [14]. (Compare the Y-shaped configuration of the homolog pair as visualized by DAPI and HTP-3 staining in Figures 2D and F with Figures 6M and N.) Disrupted bivalent differentiation was also apparent when comparing SYP-1 staining between the wild-type homolog pair (Figure 2F) and the *zhp-3::gfp* homolog pair at high temperature (Figure 6O), despite obvious regions of SYP-1 enrichment and depletion. In Figure 2F, the homologous region enriched in SYP-1 remained closely synapsed while the regions from which SYP-1 was departing were visibly separating. In Figure 6O, the regions of SYP-1 enrichment also appeared to be undergoing desynapsis, similar to the regions where SYP-1 was being ejected.

We also observed instances where the homolog pair appeared to separate as the SC disassembled in diplotene, resulting in two separate DAPI-staining bodies (6Q). Each DAPI-staining body included a region of SYP-1 enrichment (Figures 6S), indicating that a crossover did occur between these chromosomes. This suggests that some univalents observed later in meiotic prophase (Figure 5E) may arise from recombinant chromosomes with unstable chiasmata.

One prediction is that these defects in bivalent differentiation might lead to irregular bivalents at diakinesis. We visualized bivalent structure in the most proximal oocyte of the hermaphrodite germline (commonly referred to as the -1 oocyte) by DAPI staining and immunofluorescence against the axial element HTP-3 (Figures 6U–X). In wild-type nuclei, each bivalent appeared as a compact DAPI-staining body with HTP-3 highlighting a cruciform scaffold (Figure 6U). In *zhp-3::gfp* mutants at 15° (Figure 6V), 20° (Figure 6W) and 25° (data not shown), the integrity of the compact DAPI-staining body appeared compromised and the HTP-3 staining was disorganized, indicating perturbed chromosome structure. We also saw disrupted HTP-3 localization in *smo-1* single mutants in diakinesis (Figure 6X). As described above, *zhp-3::gfp* mutants at 15° and 20° had no apparent defects in SC disassembly during late pachytene and diplotene (Figure 6B and data not shown). This inconsistency suggests that there are indeed defects in SC disassembly and bivalent differentiation in *zhp-3::gfp* mutants at these lower temperatures that are not detected in our assays but that nonetheless impact bivalent formation. *zhp-3::gfp* bivalents at all temperatures showed reciprocal localization of AIR-2 and HTP-1 (data not shown). The high frequency of inviable and male progeny among the progeny of *zhp-3::gfp* animals indicates that these bivalents are not fully competent for homolog disjunction, despite the presence of chiasmata and the asymmetric recruitment of factors required for the stepwise release of cohesion [14],[32],[37].

## Discussion

### ZHP-3 Is a Cytological Marker for Crossover Formation in *C. elegans*

Our cytological characterization of ZHP-3 reveals that both the native protein and GFP fusion localize to sites of crossover recombination in late pachytene and diplotene. Thus, ZHP-3 is the first protein known to mark crossovers in *C. elegans*. The ability to directly visualize reciprocal exchange during meiosis in this organism provides an important addition to the toolbox for the analysis of meiotic events in *C. elegans* [11],[38]. This tool has enabled us to directly measure the numbers of crossovers on a per-chromosome and per-nucleus basis in oocytes, rather than in progeny, and has revealed that genetic assays may underestimate the frequency of multiple crossovers that arise in mutant backgrounds (Figure 3G).

### An Additional Role of ZHP-3 in Promoting Proper Meiotic Chromosome Segregation

Initial characterization of *zhp-3* revealed that it is required for the formation of crossovers [10]. Our data indicate that ZHP-3 plays an additional role late in meiotic prophase that contributes to proper chromosome segregation. *zhp-3::gfp* mutants exhibit elevated rates of progeny inviability and male production despite nearly wild-type levels of recombination and robust bivalent formation, indicating that the absence of crossover recombination is not responsible for the defects in chromosome segregation we observe.

One possible explanation is that the frequency of recombination we detected by genetic assays in *zhp-3::gfp* mutants may reflect selection bias imposed by the requirement for viable progeny. We think this is unlikely for several reasons. 1) The absence of meiotic recombination does not preclude the production of viable progeny. Mutant animals that fail to introduce chiasmata on all chromosomes during meiosis still produce viable progeny [10],[12],[20], probably due to random chromosome segregation of a limited number of chromosomes (six) and the ability of worms to tolerate some aneuploidy. 2) In *zhp-3::gfp* animals, oocytes exiting pachytene appear to asymmetrically disassemble their SCs, a cytological indicator for crossover completion. Even at 25°, where the embryonic lethality of progeny approaches that of the null mutant and condensed bivalents have SYP-1 on both axes, defects in SC disassembly at diplotene are subtle. 3) In addition to embryonic inviability due to autosomal aneuploidy, the Him, or “high incidence of males,” phenotype is diagnostic for a defect in meiotic chromosome segregation. Embryos that have two copies of the *X* chromosome develop as hermaphrodites, while embryos with a single *X* develop as males. Although we observed a decrease in recombination on the *X* chromosome (75% of wild-type levels in *zhp-3::gfp* at 20°), the incidence of male self-progeny (21.3%) is similar to that seen in homozygous *zhp-3* null mutants (27.8% in Table 2 and 22.2% in [10]), which had no recombination in a similar interval on the *X* chromosome [10]. In other experiments, *zhp-3::gfp* mutants exhibited more severe Him phenotypes (performed at 20°, data not shown), similar to the Him phenotypes of other *C. elegans* mutants that fail to introduce crossovers between homologs, *msh-5(me23)* and *spo-11(ok79)* [9],[20], highlighting the regularity with which *X* chromosomes missegregate despite the frequency of crossover recombination in *zhp-3::gfp* mutants.

Furthermore, the average number of bivalents present in these animals at all temperatures is inconsistent with the number of viable progeny they produce. *zim-2* mutants, in which a single pair of autosomes fails to recombine, show an average of 6.8 DAPI-staining bodies at diakinesis and produce nearly 70% viable progeny [31]. This contrasts with *zhp-3::gfp* mutants at 20°, which show approximately the same average number of DAPI-staining bodies (and almost half of the diakinesis nuclei in these animals have six bivalents), yet produce only 12.6% viable progeny. *zim-1* mutants, in which two pairs of chromosomes fail to recombine, have an average number of DAPI-staining bodies equivalent to the *zhp-3::gfp* mutant at 25° but produce 26.1% viable progeny [31]. This incongruity suggests that the bivalents that do form in *zhp-3::gfp* mutants are not functional for proper chromosome segregation. This idea is further supported by the defects in bivalent structure, as visualized by DAPI and HTP-3 staining, we observe in *zhp-3::gfp* mutants (Figures 6V and W).

In light of the defects in SC disassembly and bivalent structure that we observed in *zhp-3::gfp* mutants, we favor the hypothesis that after promoting crossover formation, ZHP-3 remains localized to the site of the crossover to regulate SC disassembly and bivalent formation to produce stable bivalents with functional chiasmata. The temperature sensitivity we observe may be explained by ZHP-3-GFP's loss of function at higher temperatures. Alternatively, it may reflect an increased requirement for ZHP-3 function at higher temperature, potentially analogous to the barrier to crossover formation in budding yeast sporulated at 33°C [2].

We interpret our data to indicate that *zhp-3::gfp* mutants have four classes of chromosome configurations during late meiotic prophase: 1) univalents which have not undergone crossing-over. These are most likely to be *X* chromosomes, given the significant reduction in recombination we observe on the *X* (Figure 4G and Table 3); 2) univalents that have undergone recombination but failed to maintain their linkage during disrupted bivalent differentiation, (Figure 6Q–T); 3) bivalents that have recombined but undergone defective bivalent differentiation, preventing their proper segregation (Figures 6M–P); and 4) functional bivalents that can be segregated correctly. With increasing temperature (or reduction of SUMO; Figure 5E), *zhp-3::gfp* is less able to coordinate crossover formation with large-scale changes in chromosome structure that accompany bivalent differentiation. Consequently, class 4 becomes more rare and classes 2 and 3 more prevalent, resulting in meiotic chromosome missegregation and an increased frequency of inviable and male self-progeny. Class 1 is likely to remain constant given the small differences in recombination frequency on both the *X* and autosomes between 15° and 20° in *zhp-3::gfp* mutants (Figure 4G and Table 3).

The inability of class 3 bivalents to segregate correctly may be structural. Defective bivalent differentiation may produce a bivalent that is topologically or structurally unable to segregate due to altered chromosome morphology. An alternate explanation may be that defective bivalent differentiation prevents the proper localization of factors required for chromosome segregation. The proper localization of AIR-2 on bivalents in *zhp-3::gfp* mutants argues against the latter explanation but since it is only one of many factors required to correctly segregate chromosomes, we cannot discount the possibility that other proteins mislocalize on these bivalents. The low penetrance of the chiasma instability phenotype (why don't all the bivalents fall apart?) may be explained by the location of crossovers: crossovers located more internally on the chromosome may have a higher likelihood of maintaining some kind of linkage, albeit an aberrant one, due to additional chromosomal constraints (e.g. topological, cohesin-mediated, etc.) [15].

ZHP-3 may promote SC disassembly and bivalent formation directly or regulate an upstream event that has repercussions for these processes. For example, crossovers at the level of the DNA duplex are thought to be accompanied by exchange at the level of chromosome axes, ensuring axial continuity during chromosome segregation [39]. The recombination-dependent destabilization of axis components, such as the cohesin Spo76/Pds5, has been shown to occur early in prophase in some fungi [40], concomitant with crossover designation. However, exchange is thought to be finalized later, perhaps not until pachytene [41]. An inability to couple genetic exchange with necessary alterations in chromosome architecture may produce defective bivalents unable to maintain chiasmata or undergo proper chromosome segregation.

### What Underlies the Dynamics of ZHP-3 Localization?

The localization of ZHP-3 changes dramatically during meiotic prophase, first along the length of the synaptonemal complex, then asymmetrically on the SC, eventually concentrating at sites of crossover recombination. This change in localization occurs after the established role of ZHP-3 in promoting crossover recombination events in the early stages of meiotic prophase [10]. We have argued above that this shift in localization reflects an additional role for ZHP-3 in late meiotic prophase. However, additional events in this stage of meiosis may also impact ZHP-3 localization.

The redistribution of ZHP-3 during pachytene coincides with a key transition point in meiotic prophase in which chromosomes crossover intermediates are fully resolved to generate chiasmata [42],[43] and any remaining DSBs are repaired by an alternate mechanism in which the sister chromatid is available as a template [44]. This suggests that the presence of ZHP-3 along the SC may be linked to the mode of DSB repair, and specifically to crossover competence. Consistent with this hypothesis, we found that the change in localization is abrogated at restrictive temperature in a MAP kinase mutant (*mpk-1(ga111ts))* (Figure S2), which disrupts this switch in the mode of recombinational repair at the end of prophase [44]. The delay in ZHP-3 relocalization in mutants with disrupted crossover control further supports a relationship between recombination status and ZHP-3 targeting. However, the asymmetric localization of ZHP-3 on the SC is unlikely to be necessary for this switch, since mutants that fail to make crossovers (*e.g.*, *msh-5(me23)* mutants) are thought to repair DSBs by this alternate pathway, and this apparently occurs without relocalization of ZHP-3 (Figure 2H and [11]).

Alternatively, the change in ZHP-3 localization might be a response to other MAP kinase-regulated events. For example, physical assays in budding yeast indicate that the resolution of double Holliday junctions (DHJs) occurs late in pachytene, just prior to SC disassembly [42],[43]. The relocalization of ZHP-3 and formation of foci may indicate the transition of these crossover intermediates into mature crossovers. The pachytene arrest seen in *mpk-1* mutants [45] makes it difficult to address the role of the MAP kinase pathway in crossover resolution. However, the timely repair of DSBs in *mpk-1* mutants indicates no obvious defect in the early events of crossover formation (data not shown).

### A Role for SUMO in the Formation of Functional Bivalents

Chromosome synapsis and recombination appeared unperturbed by cytological assays in *smo-1* and *ubc-9* mutants (Figure 5A, C, E and data not shown), indicating that these genes are dispensable for SC assembly and crossover formation in *C. elegans*. However, *smo-1* mutants showed defects in SC disassembly (Figure 6G–I) and bivalent organization (Figure 6X), and a small fraction of their diakinesis nuclei (5%) showed univalents, all phenotypes shared by *zhp-3::gfp* mutants. In addition, the incidence of univalents was further elevated in *zhp-3::gfp* mutants at all temperatures when SUMO was absent or limiting (Figure 5E), indicating that both SUMO and ZHP-3, working together or independently, promote bivalent differentiation. The identification of ZHP-3 as a potential SUMO ligase is more consistent with the idea that these genes work in the same pathway. However, it is difficult to reconcile this idea with the relatively subtle meiotic defects seen in *smo-1* mutants. One possible explanation is that homozygous *smo-1(ok359)* animals have residual SUMO function in the germline. Attempts to further reduce germline *smo-1* function by RNA interference have not resulted in a more severe meiotic phenotype (data not shown). Additional characterization of the biochemical activity of ZHP-3 and its regulation will help to determine the role of SUMO in SC disassembly and bivalent differentiation in *C. elegans*.

### Conservation of Zip3/ZHP-3 Function: Coordinating Meiotic Recombination with SC Morphogenesis

Our results lead us to propose the following model: ZHP-3 promotes crossover formation early in meiotic prophase, consistent with findings in budding yeast [1],[2] and *C. elegans* [10]. After crossover designation, ZHP-3 remains localized at the site of the crossover to restructure the bivalent and regulate chiasma function. This model suggests a conserved function for Zip3/ZHP-3 in coordinating crossover formation with SC morphogenesis. In *S. cerevisiae*, ZIP3 appears to couple crossover recombination with synapsis initiation [1]. In *C. elegans*, an organism that does not require recombination to initiate synapsis [9], ZHP-3 couples crossover formation with SC disassembly and bivalent formation. The possibility that Zip3 might also play a similar role in budding yeast has not yet been explored. The identification of ZHP-3 and/or SUMO targets on meiotic chromosomes will refine our understanding of how chromosome structure is remodeled to accompany reciprocal exchange.

## Materials and Methods

### Worm Strains, Genetics, and Culture Conditions

The wild-type *C. elegans* strain was N2 Bristol. All experiments were performed at 20° under standard conditions [46], unless otherwise noted. Mutations and rearrangements [9],[10],[20],[24],[30],[31],[35],[47] used were as follows:

Chromosome *I*: *smo-1(ok359)*, *zhp-3(jf61)*, *hT2[bli-4(e937) let-?(q782) qIs48 (I:III)]*, *mnDp66*
Chromosome *III*: *mpk-1(ga111)*, *unc-119(ed3)*, *hT2[bli-4(e937) let-?(q782) qIs48 (I:III)]*
Chromosome *IV*: *P_pie-1_::zhp-3::gfp*, *ubc-9(tm2610)*, *zim-1(tm1813)*, *him-8(mn253)*, *spo-11(ok79)*, *msh-5(me23)*, *mIs11*
Chromosome *V*: *unc-60(e723)*, *dpy-11(e224)*
*X* Chromosome: *dpy-6(e14)*, *unc-3(e151)*, *meDf2*.

*P_pie-1_::zhp-3::gfp*; *unc-119(ed3)* was generated by ballistic transformation [27] of the *K02B12.8::GFP* reporter construct (described in [10]) into *unc-119(ed3)* mutant worms. Transformed worms were identified and scored for stable transmission of the construct, indicating integration into the genome, and expression of ZHP-3-GFP. During strain construction, *P_pie-1_::zhp-3::gfp* was followed by its ability to rescue *unc-119(ed3)* or by PCR with the primers 5′ ATT/ CGA/ CCG/ TCA/ GCA/ GAT/ AC 3′ and 5′ ATC/ TGG/ GTA/ TCT/ CGA/ GAA/ GC 3′. The *zhp-3(jf61)* allele was followed by PCR with the primers 5′ TGC/ TTCA/ ACC/ GAA/ AAC/ CAC/ C 3′, 5′ TGA/ ACC/ ACT/ TTC/ TGA/ GAG/ CC 3′ and 5′ ATT/ GTT/ TGT/ CGC/ CAA/ CCC/ TG 3′ and the *smo-1(ok359)* allele was followed by PCR with the primers 5′ TGA/ TGA/ CGG/ TTA/ AGG/ AGG/ TC 3′ and 5′ GAG/ AAG/ GTC/ ATC/ GAA/ TCT/ CG 3′. *ubc-9(tm2610)* was generated by the National Bioresource Project for the Nematode and backcrossed to wild-type animals three times before analysis.

Recombination assays were performed by crossing *zhp-3(jf61)/hT2*; *unc-119(ed3)/hT2*; *P_pie-1_::zhp-3::gfp* males with *zhp-3(jf61)/hT2*; *P_pie-1_::zhp-3::gfp*; *unc-60(e723) dpy-11(e224)* or *zhp-3(jf61)/hT2*; *P_pie-1_::zhp-3::gfp; dpy-6(e14) unc-3(e151)* hermaphrodites. NonUnc, nonDpy F1 cross progeny homozygous for *zhp-3(jf61)* were picked and allowed to self-fertilize at the relevant temperatures. Their progeny were scored for *R*, the fraction of recombinant (Dpy nonUnc and Unc nonDpy) progeny. However, to avoid the possibility of including triplo-*X* animals arising from *X* chromosome missegregation as Dpy nonUnc recombinants, R was calculated by doubling the number of Unc nonDpy progeny. Map distances (*p*) were calculated using the equation *p* = (*1−(1−2R)^1/2^*)×100. For frequency of recombination on the *X* chromosome, map distance was calculated as in [20].

### Antibodies, Immunostaining, and DAPI Analysis

To raise antibodies against ZHP-3, a 214 amino acid polypeptide corresponding to the C-terminus of ZHP-3 was cloned into a bacterial expression vector. The primers 5′ CAC/ CTC/ TCA/ AAC/ TCC/ ATT/ TCC/ ATT/ CA 3′ and 5′ TTA/ ATC/ GGC/ GGG/ TCC/ AAT/ GA 3′ were used to amplify the sequence from cDNA clone yk703a9, kindly provided by Yuji Kohara (National Institute of Genetics, Mishima, Japan). The resulting PCR product was cloned into the pENTR/D-TOPO vector (Invitrogen). Individual clones were sequenced and a correct clone was used in a Gateway LR Clonase II enzyme (Invitrogen) reaction to recombine the insert into pDEST15 (Invitrogen) to generate an N-terminal tagged GST-fusion protein. This plasmid was transformed into *E. coli* BL21 DE3. Recombinant protein was purified according to [48] and used to immunize guinea pigs. Antiserum containing anti-ZHP-3 was used for in situ cytological experiments at 1∶500 dilution. The specificity of the antibody was verified by the absence of all reported staining patterns in *zhp-3* null mutants (data not shown).

For immunofluorescence, Late L4 stage worms were picked and allowed to age 20–24 hours. Gonad dissection was carried out in 1× EBT (25 mM HEPES-Cl pH 7.4, 118 mM NaCl, 48 mM KCl, 2 mM EDTA, 0.5 mM EGTA, 0.1% Tween 20, 0.5 mM spermine, 0.15 mM spermidine)+20 mM sodium azide. An equal volume of 2% formaldehyde in EBT (final concentration is 1% formaldehyde) was added and allowed to incubate for five minutes. The sample was freeze-cracked and incubated in methanol at −20° for one minute and transferred to PBST. The remainder of the immunostaining protocol was as in [49]. Additional antisera included monoclonal mouse anti-GFP (Q-Biogene), polyclonal rabbit anti-GFP conjugated to Alexa-Fluor 488 (Invitrogen), polyclonal rabbit and guinea pig anti-SYP-1 [12] and polyclonal chicken and guinea pig anti-HTP-3 [50]. Secondary antibodies were purchased from Jackson ImmunoResearch and Molecular Probes. All antibodies were diluted 1∶500 for immunostaining.

To maximize the number of nuclei in diakinesis for analysis of DAPI-staining bodies, late L4 stage worms were picked and allowed to age for 36 hours at 25°, 48 hours at 20° or 72 hours at 15°. Gonad dissection was as described above, except an equal volume of 7.4% formaldehyde in EBT (final concentration is 3.7%) was added and allowed to incubate for five minutes.

## Supporting Information

Figure S1 Changes in crossover distribution can be visualized by localization of ZHP-3 foci. A. Location of a ZHP-3 focus toward one end of a pair of synapsed homologs in wild-type animals. B. *rec*-1 mutants eliminate this bias. Meiotic nuclei in both genotypes were stained with antibodies against ZHP-3 and SYP-1. Scale bar indicates two microns.
(0.76 MB EPS)

Figure S2 ZHP-3 remains localized along the SC in late pachytene in *mpk-1* mutants. Late pachytene meiotic nuclei in wild-type (A–C) and *mpk-1* (*ga111*) mutants (D–F) stained with antibodies against ZHP-3 and SYP-1. Scale bar indicates four microns.
(1.88 MB EPS)
