# Correction: ZHP-3 Acts at Crossovers to Couple Meiotic Recombination with Synaptonemal Complex Disassembly and Bivalent Formation in *C. elegans*

**DOI:** 10.1371/annotation/ffbb52bd-7ceb-404c-8c96-93577bf83932

**Published:** 2008-11-08

**Authors:** Needhi Bhalla, David J. Wynne, Verena Jantsch, Abby F. Dernburg

There was an error in Figure 6. Panels O and S were duplicated. The correct version of Figure 6 is available here:

**Figure 6:**
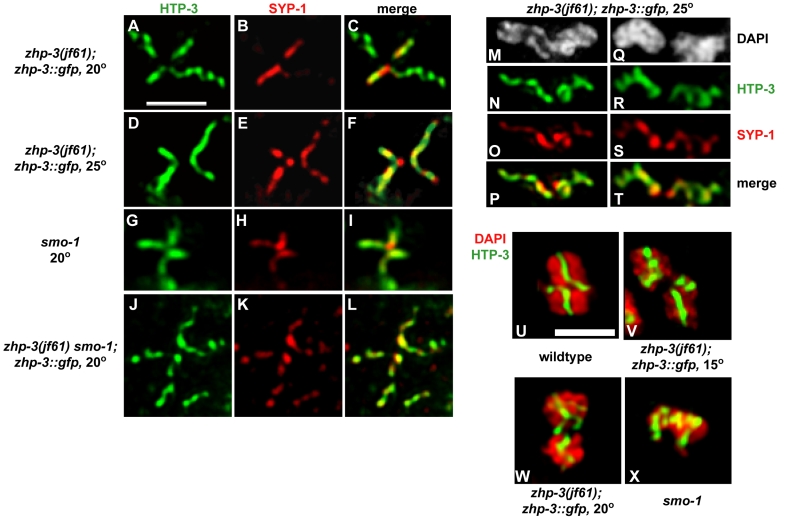
Defects in SC disassembly and bivalent structure in *zhp-3::gfp* mutants.

